# Commentary on “A Pilot Study Investigating the Influence of Dietary Boron Levels on Osteoporosis in Postmenopausal Women”

**DOI:** 10.1002/fsn3.4646

**Published:** 2024-12-11

**Authors:** Bahar Ullah

**Affiliations:** ^1^ Saidu Medical College Saidu Sharif, Swat Khyber Pakhtunkhwa Pakistan

## Abstract

This letter discusses the potential influence of dietary boron on osteoporosis in postmenopausal women, a demographic significantly affected by this condition. Recent studies suggest that boron plays a critical role in bone health by modulating mineral metabolism and hormonal balance. Despite its potential benefits, boron is often overlooked in dietary recommendations for this group. This correspondence aims to raise awareness and encourage further research into the incorporation of boron‐rich foods to enhance bone density and overall health in postmenopausal women.

To the Editor,

I recently come across the study titled “A Pilot Study Investigating the Influence of Dietary Boron Levels on Osteoporosis in Postmenopausal Women,” published in Food Science and Nutrition (2024) by Rababah et al., addresses a crucial area of research on bone health in postmenopausal women. The study's focus on dietary boron is particularly relevant as osteoporosis is a growing concern among aging women. While the authors provide valuable insights, I believe further discussion is warranted regarding the biochemical interplay between boron and other micronutrients essential for bone health.

## Main Points of the Study

The study found a strong positive correlation between dietary boron intake and bone mineral density (BMD) in the target population, suggesting that boron could potentially serve as an adjunct dietary factor in managing osteoporosis. This finding aligns with previous research by Nielsen (2014), which showed that boron plays a significant role in bone mineralization and bone health. However, a negative correlation between boron and serum calcium levels was also reported, raising questions about boron's interaction with calcium homeostasis.

## Critical Analysis

Hunt and Herbel (1997) have demonstrated that boron can influence both calcium and magnesium metabolism, suggesting that the observed decrease in serum calcium might not indicate a negative effect but rather an adaptive response in mineral balance. It would be beneficial for future studies to examine how dietary calcium intake, alongside boron, modulates bone health to avoid potential misinterpretations.

Furthermore, the study did not find significant associations between boron intake and other health markers, such as serum vitamin D, BMI, and physical activity. This suggests that boron's effect might operate through distinct biochemical pathways independent of these variables. To clarify this, I suggest examining boron's relationship with other micronutrients like magnesium and vitamin K, both known to be critical for bone density and structural integrity (Devirian and Volpe 2003).

## Recommendations for Future Research

Given the potential of boron as a dietary intervention, it is essential to conduct larger‐scale, randomized controlled trials to establish optimal dietary boron levels for postmenopausal women. This could help validate boron's role in bone health and provide clearer dietary recommendations.

## Conclusion

The study by Rababah et al. (2024) is a commendable initial exploration into the impact of dietary boron on bone health. Further investigation into boron's interactions with other nutrients and its long‐term effects is necessary to better inform dietary strategies for osteoporosis prevention and management.
